# Sclerosing Variant of the Bronchioloalveolar Carcinoma: Imaging Findings in an Atypical Case

**DOI:** 10.1155/2010/361265

**Published:** 2010-06-24

**Authors:** Carolina Lamas Constantino, Edson Marchiori, Gláucia Zanetti, Antonio Muccillo, Mariana Leite Pereira, Guilherme Abdalla, Pedro Martins, Nina Ventura, Rodrigo Canellas, Viviane Brandão, Romulo Varella de Oliveira

**Affiliations:** Department of Radiology, Rio de Janeiro Federal University, Rua Thomaz Cameron, 438. Valparaiso, Petrópolis, 25685.120, Rio de Janeiro, Brazil

## Abstract

Bronchioloalveolar carcinoma remains one of the most enigmatic lung cancers, demonstrating varied growth patterns, mixed histological features, and confusing clinical manifestations. This paper reports a case of an unusual form of presentation: a sclerosing type associated with desmoplastic reaction and cicatrization. A 75-year-old woman was admitted with persistent dry cough and progressive dyspnea. Physical examination showed bilateral inspiratory crackles. A chest radiograph and high-resolution computed tomography demonstrated confluent airspace nodules, forming areas of consolidation in both lungs, with signs of architectural distortion. The lung biopsy revealed a nonmucinous sclerosing bronchioloalveolar carcinoma.

## 1. Introduction

There has been a substantial increase in the percentage of pulmonary adenocarcinoma accompanied with a decrease in squamous cell carcinoma in recent decades [1]. Bronchioloalveolar carcinoma (BAC) represents 1.5%–6.5% of all primary pulmonary neoplasms. Most patients are between 40 and 70 years of age [2].

BAC is derived from the epithelial cells located distally to the terminal bronchioles, and is defined as a primary lung cancer in peripheral locations. It is found growing in a lepidic (scale-like) manner near the alveolar septae without parenchymal, vascular, or pleural invasion [3]. BAC has a distinct clinical, pathological, and radiological presentation compared to the other subtypes of adenocarcinoma. In the majority of cases, BAC appears as a peripheral pulmonary nodule; however, the tumor may present as a segmental, lobar consolidation, with a multifocal or diffuse pattern [3, 4]. In this report, we describe a case of BAC in a 75-year-old woman with an unusual presentation: a nonmucinous sclerosing type associated with desmoplastic reaction and cicatrization.

## 2. Case Presentation

A 75-year-old woman presented with a persistent dry cough that had been present for 2 months together with progressive dyspnea, anorexia, fatigue, malaise, and a weight loss of 5 kg during the same period. She did not have a fever, chills or sweats. There was no history of smoking, alcohol abuse or primary malignancy. Physical examination showed the patient to be ill-looking, acyanotic, eupneic, and afebrile. Her blood pressure was 100/60 mmHg, her pulse rate was 80 beats per minute, her respiratory rate was 24 breaths per minute, and her body temperature was 36.4°C. She had inspiratory crackles in the bilateral lower lung fields. Laboratory tests showed normal RBC and WBC count, serum creatinine was 0.6 mg/dL, urea was 23 mg/dL, glucose was 102 mg/dL, and liver function tests were normal.

**Figure 1:**
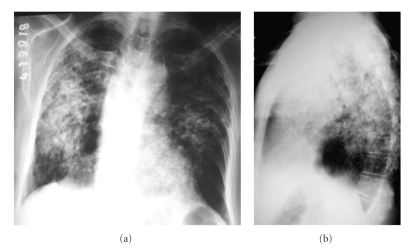
Chest radiographs in anteroposterior (a) and lateral incidence (b) showing hyperinflated lungs, with bilateral nonhomogeneous opacities, predominantly in the middle third of right lung.

**Figure 2:**
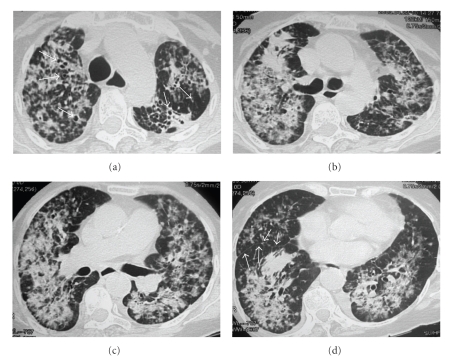
HRCT scans at four levels (a)–(d) demonstrate confluent airspace nodules and areas of consolidation in both lungs, with signs of architectural distortion that are characterized by bronchial dilatations within the lesions (white arrowheads in (a)) and fissure distortion with loss of the regularity (white small arrows in (d)).

**Figure 3:**
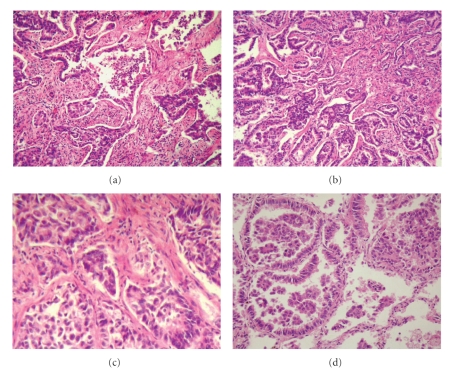
(a)–(c): Photomicrographs demonstrate active young fibroblast proliferation (fibrosis) and tumor cells that are entrapped within the central desmoplasia. (d): Photomicrograph shows neoplastic cells spreading along alveolar septa (lepidic growth) with preservation of the alveolar architecture (hematoxylin-eosin stain).

A chest radiograph revealed bilateral nonhomogeneous opacities, predominantly in the middle third of the right lung (Figure 1). The high-resolution computed tomography (HRCT) demonstrated confluent airspace nodules, forming areas of consolidation in both lungs, with signs of architectural distortion (Figure 2). Bronchoscopy showed no abnormalities. Microscopical examination and cultures of sputum and bronchoalveolar lavage were negative for mycobacteria and fungi. A cytological evaluation of a sputum specimen revealed no malignant cells. The lung biopsy revealed nonmucinous sclerosing BAC (Figure 3).

The oncologists chose palliative treatment with clinical supports and analgesia due to the advanced state of the disease. Since her performance status was ECOG 4, chemotherapy could not be used. She died one month after the admission with respiratory failure.

## 3. Discussion

This paper reports a case of BAC, which is an uncommon type of bronchial carcinoma that occurs most frequently among nonsmokers, women, and Asians [2]. According to the revised World Health Organization (WHO) lung tumor classification, BAC is defined as a subtype of adenocarcinoma with intra-alveolar spread and lepidic growth along an intact interstitial framework, yet with no evidence of stromal, vascular or pleural invasion [5]. The distinguishing feature of this carcinoma is the preservation of the underlying lung architecture. BAC tends to spread through the airways, but lymphatic and hematogenous dissemination may occur in 50% to 60% of cases [2, 6]. Though a tumor resection might prove helpful in other cases, complete resection of the tumor could only confirm the diagnosis of pure BAC since some other invasive adenocarcinomas also contain the BAC component. WHO classification is very useful in differentiating BAC from other tumors with BAC components. Despite appropriate classifications, patients seek medical care once the cancer has substantially advanced or is metastatic. In the majority of these cases complete resection is not feasible [7]. Similarly, our patient presented with a multifocal advanced disease.

BAC is classified into three different subtypes: mucinous, nonmucinous, or mixed (including both types) [8]. In the sclerosing form of nonmucinous BAC, the intratumoral alveolar septa, while intact, are thickened by fibrosis and inflammation. In addition, there is dense central sclerosis, which entraps and distorts the linearly spreading tumor cells, giving the appearance of invasive glands [9]. Our case is an interesting example of nonmucinous sclerosing BAC, which demonstrated a diffuse and multicentric growth pattern with desmoplastic reaction. While a biopsy is focal, the use of imaging can confirm whether a diffuse alveolar infiltrate is present in both lungs. The HRCT pattern was homogeneous and the same pattern was seen in all the pulmonary lobes. Based on these results, one can infer that the histological findings would also be similar.

More than half of all patients with BAC are asymptomatic and remain without symptoms even as the disease disseminates. The most frequently reported symptoms and signs are cough, sputum, shortness of breath, weight loss, hemoptysis, and fever. Bronchorrhea is unusual and is a late manifestation seen only with diffuse BAC [2]. The clinical presentation of our patient was similar to that in previously documented cases.

There are three different radiological patterns seen in BAC: a solitary nodule or mass of varying density, focal consolidation, or multifocal (diffuse) disease [5]. The most common radiological finding is a solitary peripheral, lobulated or ill-marginated pulmonary nodule or mass. Other CT features include pseudocavitation (bubble-like low attenuating region within a nodule), heterogeneous attenuation, and irregular margins forming a star pattern and pleural tags, which are thought to be caused by a desmoplastic reaction in the peripheral septa of the lung [10]. Focal isolated ground-glass opacity has been described as an early finding of BAC, especially in cases where there are associated pseudocavities and air bronchograms [5].

The consolidative form of BAC (30% of cases) corresponds to a mucinous histological subtype, and may be focal, ill-defined, ground-glass, lobar or multifocal. Because lobar consolidation can be difficult to differentiate from pneumonia in plain radiography images, the diagnosis is often delayed. Evaluation by CT provides additional information needed for diagnosis. Nonresolving central or peripheral consolidation, especially with associated nodules, raises the possibility of BAC. The nodules are predominantly centrilobular, reflecting an endobronchial distribution. Peribronchial nodules may represent lymphatic spread. As the tumor fills the alveolar spaces and infiltrates the alveolar septa and bronchial walls, the bronchus becomes narrowed, stretched and rigid. Bulging of the interlobar fissure is a characteristic finding in consolidative BAC, and is probably a result of mucin production in the tumor, resulting in swelling of the lobe [6, 11].

If contrasted CT is performed, the “CT angiogram" sign can be seen. This sign represents the enhancement of unaffected pulmonary vessels coursing through low-attenuation consolidated lung parenchyma, filled with mucus and fluid. This feature is not specific, and may be seen in BAC, obstructive pneumonitis, pneumonia, pulmonary lymphoma, and lipoid pneumonia [12]. High-resolution CT findings of diffuse BAC include ground-glass opacity (most common), consolidation, nodules, centrilobular nodules, peripheral distribution, air bronchogram, and lower lung predominance. The diffuse nodular form is often indistinguishable from miliary tuberculosis or metastatic thyroid carcinoma [6]. Extensive ground-glass airspace opacification associated with septal thickening (the ‘‘crazy paving” pattern) has also been described [13].

The chest radiograph of our patient showed nonhomogeneous opacities and the HRCT revealed a pattern of consolidation and airspace nodules. The HRCT also demonstrated lung architectural distortion that was characterized by bronchial dilatations within the lesions and fissure distortion with loss of the normal regularity. Given the diffuse pattern of airspace occupation that occurred with a chronic course (two months), the main diagnostic hypotheses were infectious diseases (especially tuberculosis and fungal diseases) and neoplastic diseases (especially bronchioloalveolar carcinoma and lymphoma). The absence of fever and the laboratory test results reduced the probability of there being an infectious disease. Furthermore, bronchoalveolar lavage and sputum culture were negative for mycobacteria and fungi, making neoplastic etiology a more probable cause. The pulmonary biopsy revealed nonmucinous bronchioloalveolar carcinoma, with evidence of associated fibrosis (sclerosing component).

The prognosis of this disease depends on the stage, based on the nature and extent of the spread of the neoplasm. A localized area of segmental or lobar consolidation represents a better prognosis than does the diffuse disease [2, 4]. Although the grade of fibroblastic proliferation and fibrotic scarring (central fibrosis) are important prognostic factors for bronchioloalveolar carcinomas [14–16] in patients with advanced BAC, death typically occurs from respiratory failure secondary to diffuse pulmonary involvement rather than from spread of the disease to other organ sites. This is attributed to the fact that BAC is characterized by diffuse alveolar filling, which frequently results in hypoxemic respiratory failure [14, 17]. In our patient, the rapid evolution to death was most likely the result of extension of the lesions, with diffuse airspace occupation.

Surgical resection is the only potentially curative treatment; however, multifocal diseases remains problematic, as treatment options are limited. One multicenter review of lung transplantation for multifocal BAC reported a 5-year survival rate of 39%. Although it is rarely curative, lung transplantation remains a valuable option for patients with impending respiratory failure secondary to advanced multifocal BAC [18].

In conclusion, BAC may present with a variety of CT appearances and is associated with variable prognoses depending upon whether the diseased tissue is focal or diffuse. Features such as the CT angiogram sign or air bronchograms in solitary nodules and in the periphery of larger consolidations may lead the radiologist to determine the diagnosis. Persisting pure ground-glass opacities, unresolving consolidation, and a combination of diffuse nodules and consolidation found in these tests should alert the radiologist to consider a diagnosis of BAC [5]. Ultimately, sclerosing BAC must be considered when CT scans reveal architectural distortion that anatomopathologically corresponds to desmoplastic reaction and fibrosis.
